# Recent research progress of circular RNAs in hepatocellular carcinoma

**DOI:** 10.3389/fonc.2023.1192386

**Published:** 2024-01-23

**Authors:** Zhi-di Li, Yang-ling Li, Jing Lu, Shang Liang, Chong Zhang, Ling-hui Zeng

**Affiliations:** ^1^ Key Laboratory of Novel Targets and Drug Study for Neural Repair of Zhejiang Province, School of Medicine, Hangzhou City University, Hangzhou, Zhejiang, China; ^2^ Department of Pharmacology, Zhejiang University, Hangzhou, Zhejiang, China; ^3^ Department of Clinical Pharmacology, Key Laboratory of Clinical Cancer Pharmacology and Toxicology Research of Zhejiang Province, Affiliated Hangzhou First People’s Hospital, Zhejiang University School of Medicine, Hangzhou, Zhejiang, China

**Keywords:** CircRNAs, hepatocellular carcinoma, carcinogenesis, progression, biomarker

## Abstract

Hepatocellular carcinoma (HCC) is an extremely heterogeneous malignant tumor with a high morbidity and mortality. Circular RNAs (circRNAs) are noncoding RNAs with high stability, organ/tissue/cell-specific expression and are conserved across species. Accumulating evidence suggested that circRNAs play crucial roles as microRNA sponges, protein sponges, scaffolds, recruiters and could even polypeptide encoders. Many studies have since revealed that circRNAs were aberrantly expressed in HCC and acted as crucial modulators of HCC carcinogenesis and progression. Furthermore, circRNAs have also been identified as potential diagnostic and prognostic biomarkers for HCC. In this review, we thoroughly outline and evaluate the function of circRNAs in HCC development, with an emphasis on the specific molecular pathways by which they participated in the formation and progression of HCC, and we address their potential for serving as clinical biomarkers in HCC.

## 1 Background

Hepatocellular carcinoma (HCC) is one of the most prevalent primary liver cancers with a poor prognosis and is the fourth leading cause of cancer-related death worldwide (1). Currently, the principal treatments for HCC are surgical resection along with radiotherapy and chemotherapy. However, postoperative recurrence, metastasis and acquired chemoresistance strongly reduce the therapeutic efficacy of the aforementioned treatments (2). Because the carcinogenesis and progression of HCC are exceedingly complex processes, the mechanisms underlying the development of HCC remain poorly understood. Although mounting evidence compelling demonstrated that numerous noncoding RNAs (ncRNAs) play prominent roles in a wide range of clinical and physiological processes, including the progression of HCC, the function of circRNAs remains to be clarified.

CircRNAs, a group of ncRNAs, are distinguished by covalent closed-loop structures without terminal 5’ and 3’ poly(A) tails, which are generated by a special noncanonical splicing mechanism known as back-splicing (3). CircRNAs are more stable than linear RNA because of their unusual architecture, which protects them from exonuclease degradation. In addition, circRNAs are characterized with organ/tissue/cell-specific expression and are conserved across species. There is growing evidence showed that aberrantly expressed circRNAs exert crucial effect on HCC cell proliferation, apoptosis, metastasis and chemoresistance. Moreover, circRNAs can be identified as non-invasive biomarkers because they can be transmitted into the blood by exosomes. Due to the lack of obvious symptoms at the early stage and lack of dependable and valid biomarkers, HCC patients are frequently diagnosed at the advanced stage and the overall survival rate is less than ideal. As a result, an increasing number of studies have focused on the potential function of circRNAs in HCC as noteworthy disease biomarkers. In this study, we extensively describe the circRNAs involved in the progression of HCC and recent clinical research findings associated to these circRNAs. Hopefully, this comprehensive review of HCC-associated circRNAs could help to improve understanding of complex mechanisms and provide valuable clues for future HCC therapy and diagnosis strategies.

## 2 The biogenesis of circRNAs

CircRNAs, discovered in the 1990s, are a novel class of endogenously produced ncRNAs that were previously assumed to be the result of aberrant splicing. Unlike lncRNAs, which have 5′-end m7G caps and 3′-end poly(A) tails (4), circRNAs are covalent closed-loop structures without terminal 5’ and 3’ poly(A) tails that are produced from introns or exons via back-splicing (5). These characteristics are linked to numerous properties, including high abundance, stability, special conservation, and organic/tissue/cell-specific expression patterns, indicating that circRNAs extensively participate in a wide variety of physiological and pathophysiological processes (6).

CircRNA closed loop structures are formed via three main circularization mechanisms: 1) intron pairing, inverted complementary intronic sequences (such as Alu elements) flanking the exon junction could form a loop which bringing the 3’ extremity of a downstream exon (donor) proximity to the 5’ extremity of an upstream exon (acceptor), and thus facilitate the circRNAs looping structure (7). 2) RNA-binding proteins (RBPs)-mediated procedures, for example, the RBP Quaking (QKI) and FUS can recognize and interact with specific flanking intronic motifs, which driving back-splicing by direct interaction or dimerization (8). and 3) lariat intron-driven ways, during conventional splicing event, the skipped exonic and intronic sequences are integrated into a circular lariat by ‘head-to-tail’ junction, which can be processed again to form circRNAs (9). CircRNAs are classified into three types based on their origins and internal elements: exonic circRNAs (EcircRNAs), exon-intron circRNAs (EIciRNAs), and intronic circRNAs (ciRNAs). EcircRNAs are chiefly situated in the cytoplasm, whereas ciRNAs and EIciRNAs have higher content in the nucleus (10).

## 3 Biological functions of circRNAs

Accumulating studies indicated that circRNAs may play a vital role in various biological processes, including miRNA sponging, protein translation, RBP interactions, as well as other unknown processes.

### 3.1 CircRNAs act as sponges of miRNAs

CircRNAs harbor well-conserved nucleotides and canonical miRNA response elements (MREs), implying that some circRNAs can function as miRNA sequesters or sponges (11). CiRS-7 also referred to as cerebellar degeneration-related protein 1 transcript (CDR1as) and was the first EcircRNA acted as miRNA sponges. CiRS-7 contains over 70 miR-7 binding sites and can dramatically impair miR-7 activity (9). According to recent evidence, many functioning circRNAs have the latent capacity to operate as miRNA sponges and are related to HCC progression (**Supplementary Table 1**). These findings directly demonstrated that miRNA sponging is a common function for circRNAs. However, most circRNAs contain few binding sites for miRNAs, therefore it is unclear how circRNAs exert substantial effects.

### 3.2 CircRNAs bind with RBPs

Aside from binding miRNAs, circRNAs have been shown in a few studies to combine with several RBPs to form RNA-protein complexes that affect gene expression and ultimately influence the progression of various disorders. For instance, circZKSCAN1 was recently shown to be a suppressive cancer stem cells (CSCs) modulator via interacting with fragile X mental retardation protein (FMRP) rather than serving as a miRNA sponge (12). However, not all circRNAs bind to proteins and inhibit protein function. CircRHOT1, For example, has been discovered to accelerate HCC growth and metastasis by recruiting Tat-interacting protein 60 (TIP60) to upregulate nuclear receptor subfamily 2, group F, member 6 (NR2F6) expression (13). Interestingly, circRNA-SORE, a novel circRNA, played a momentous role in the maintenance and spread of sorafenib resistance in HCC by interacting with Y-box binding protein 1 (YBX1) to change its subcellular localization and sequentially preventing PRP19-mediated YBX1 ubiquitination and degradation (14). In the cases above, circRNAs acted as protein decoys, scaffolds, and recruiters in the progression of HCC. Nevertheless, bioinformatic analyses suggested that circRNAs had a lower abundance of RBP binding sites than linear RNAs, and aside from their mutual binding sites and/or sequences, the exact regulatory mechanisms connecting circRNA with RBPs and altering protein-protein interactions remain unclear and require further investigations.

### 3.3 CircRNAs encode proteins

Although circRNAs are classified as a type of ncRNA, there is growing evidence that some circRNAs are translatable. A recent study discovered that circβ-catenin was translatable and highly expressed in HCC, and translation of circβ-catenin generated a novel isoform named β-catenin-370aa that promoted HCC progression by protecting β-catenin from GSK3β-induced degradation and thus indirectly facilitating activation of the Wnt/β-catenin pathway (15). Another study revealed that circMRPS35 exerted its oncogenic function in HCC not only by sponging miR-148a to modulate the STX3-PTEN pathway, but also by being translated into circMRPS35-168aa, which resulted in cisplatin resistance, indicating that circMRPS35 might be a vital component in the progression and chemoresistance of HCC with different expression patterns under different conditions (16). The protein-coding function of circRNAs is a novel aspect in the development of diseases, and the function of protein-coding circRNAs may be more crucial than previously believed. Nevertheless, the regulatory frameworks of circRNA translation processes have received little attention, and whether circRNA derived proteins have discernible effects on the development of HCC remains controversial.

## 4 CircRNAs and HCC

**Figure 1:**
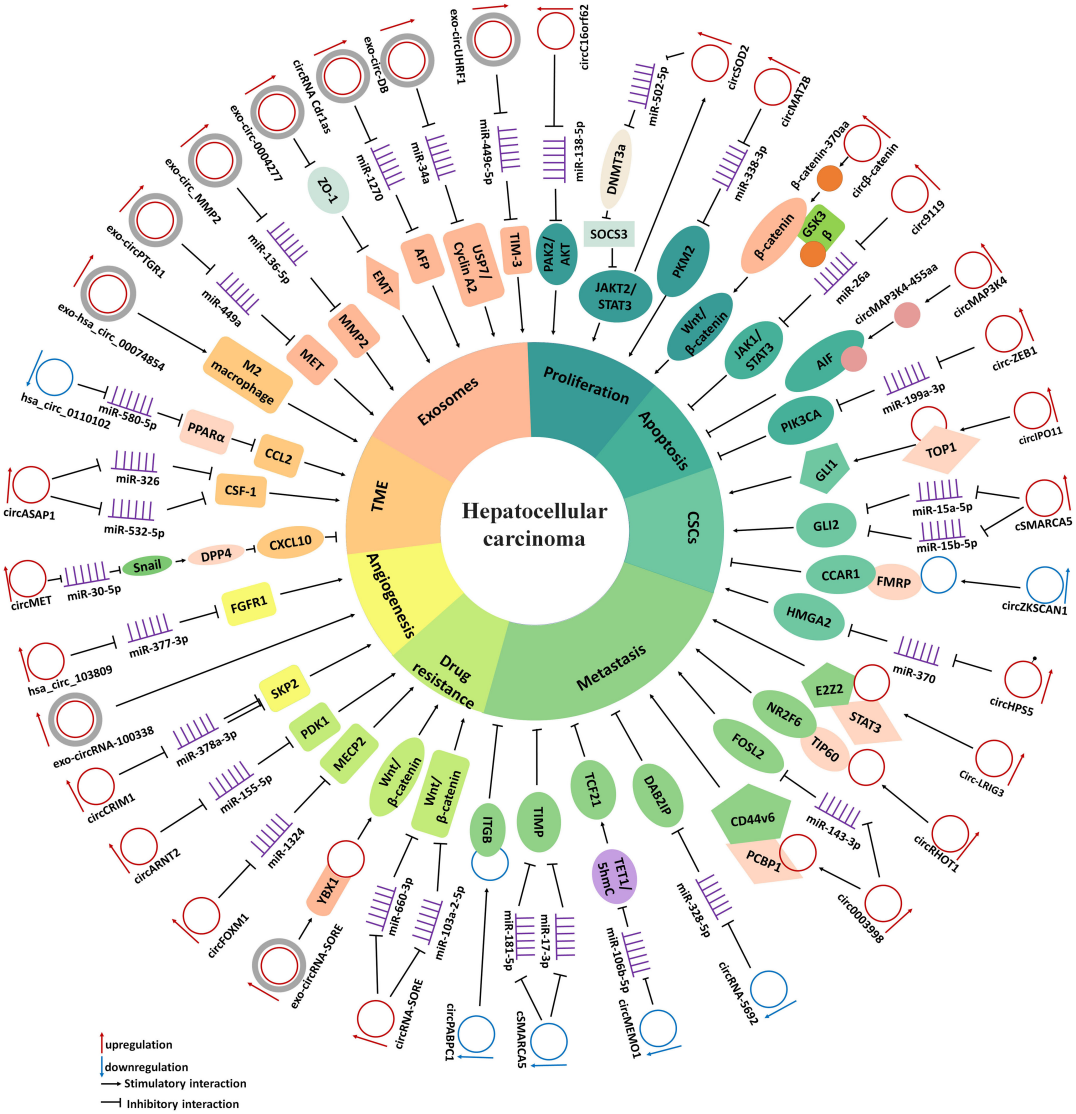
The diagram demonstrates the mechanism by which circRNAs in the regulate cell proliferation, apoptosis, cancer stem cells, metastasis, drug resistance, angiogenesis, tumor microenvironment and exosomes in HCC.

With the continuous improvement of detection technologies, a large number of circRNAs have been validated as considerable molecular regulators in the development of HCC. **Supplementary Table 1** and **Figure 1** list the expression of aberrant circRNAs in HCC along with their roles. These circRNAs are essential for several processes associated with HCC, such as cell proliferation, apoptosis, CSCs, metastasis, epithelial-mesenchymal transition (EMT), chemoresistance, angiogenesis, and have effects on the tumor microenvironment (TME), exosomes and other factors. Thus, we discussed these dysregulated circRNAs and their molecular and pathogenic mechanisms in HCC.

### 4.1 Cell proliferation

Tumor cells maintain proliferative conditions abnormally active by stimulating cell proliferation signaling pathways. The PI3K/AKT/mTOR axis is a crucial pathway in the advancement of HCC, affecting a wide range of malignant progression (17). The m6A-modified circMDK regulated the miR-346/miR-874-3p-ATG16L1 axis, activating the PI3K/AKT/mTOR signaling pathway and eventually accelerating the process of HCC carcinogenesis (18). Circ-CDYL was reported to influence the HDGF-NCL-PI3K-AKT and HIF1AN-NOTCH2 pathways as well as boost SURVIVIN and C-MYC expression, promoting stem-like characteristics and cell proliferation in HCC (19). Moreover, circC16orf62 sustained the proliferation of HCC cells through the circC16orf62/miR-138-5p/PTK2/AKT regulatory network (20). Because it regulated mTOR signaling by activating the miR-141-3p/RHEB axis, circRNA-100338 was strongly linked to the stimulation of HCC cell proliferation and metastasis (21). Unexpectedly, circSOD2 played a crucial role in accelerating HCC development via the circSOD2/SOCS3-JAK2/STAT3/circSOD2 innovative feedback pathway (22). Furthermore, circMTO1 competitively sponged miR-541-5p and decreased ZIC1 expression, inhibiting HCC progression by blocking the signaling pathway of Wnt/β-catenin (23). The Hippo pathway is a highly conserved pathway that takes a pivotal part in controlling stem cell self-renewal, cell proliferation and EMT by stimulating the downstream transcriptional factors YAP and TAZ. CircCPSF6 functioned as a novel m6A-modified circRNA accelerating tumorigenicity and cell metastasis of HCC by competitively binding to PCBP2, thus weakening the PCBP2-induced destabilization of YAP1 and triggering its expression, further activating its downstream cascade (24).

Glucose metabolism reprogramming, which is a generally recognized feature of many malignancies, happens to meet the greater rate of glycolysis required for macromolecular synthesis and to support rapid proliferation in hypoxia. CircMAT2B facilitated the progression of HCC by accelerating glycolysis through regulation of the miR-338-3p/PKM2 signaling pathway under hypoxia (12). Moreover, circRPN2 played a vital role in restraining the glycolytic reprogramming, progression and metastasis of HCC by directly interacting with ENO1 to facilitate its degradation and regulating the miR-183–5p/FOXO1 signaling pathway (25). Through its interaction with YTHDF1, circRHBDD1 accelerated the translation of PIK3R1 by modifying m6A, hence facilitating the glycolysis of HCC cells (26). Lipid metabolism is also essential for cancer cells and plays a vital role in adapting tumors to the local microenvironment. As the primary modulator of lipid metabolism, Peroxisome proliferator-activated receptor-α (PPARA) was deemed to be a promising HCC treatment. Hsa_circ_0098181 engaged in the miR-18a-3p/PPARA signaling pathway to exert anti-HCC effects (27). By physically attaching to CAPRIN1 and G3BP1, circVAMP3 exhibited tumor suppressor qualities in HCC by causing CAPRIN1 to phase separate and promoting the production of stress granules, which prevented the translation of c-Myc (28).

One factor that leads a normal cell to become malignant is dysregulation of the cell cycle machinery. The important cell cycle-related proteins known as Cyclin-dependent kinases 4 and 6 (CDK4/6) are essential for controlling the G1/S transition and the advancement of the G1 phase (29). As a miR-200a-3p sponge, circ-ZEB1.33 was a tumor promotor that stimulated CDK6 expression and HCC cell proliferation (30). Moreover, by controlling the cell cycle through the miR-1263/CDK6 signaling pathway, circERBIN accelerated the growth of HCC and subsequently accelerated the G1/S transition (31). In addition, by modifying the miR-486/CDK4 axis, hsa_circ_0016788 improved the cell cycle aberrations and migratory capabilities of HCC cells (32). CircSP3 promoted HCC growth by sponging miR-198 and upregulating CDK4 (33). Furthermore, it was showed that circCCNB1 silencing suppressed GPM6A expression to participate in the cell cycle regulation of HCC cells by upregulating DYNC1I1 expression and triggering the AKT/ERK signaling pathway (34). P21 takes a crucial part in growth arrest by inhibiting the activity of cyclin D-CDK2/4 complexes. For instance, circMTO1 functioned as a critical tumor suppressor by facilitating cell cycle arrest via the miR-9/p21 pathway (35).

In addition to stimulating cell proliferation, tumor cells frequently escape the growth-inhibitory effects of tumor suppressor pathways. PTEN functions as an important tumor suppressor by restraining cell growth and proliferation. CircHIAT1 restrained HCC cell proliferation by acting on the miR-3171/PTEN axis (36). MAPK14 could limit cell growth and cancer by engaging on cell cycle checkpoints. CircSETD3 had been identified as a tumor suppressor that suppressed the tumorigenesis of HCC by modulating the miR-421/MAPK14 pathway (37). In addition, cellular senescence is a stress-mediated, persistent cell cycle arrest of cells that were previously able to replicate (38). The p53/p21 pathway is an important pathway in senescence. CircLARP4 decreased cell proliferation and induces cell cycle arrest and senescence by suppressing miR-761, thereby increasing the RUNX3 expression and regulating activation of the downstream p53/p21 signaling pathway (39). In addition to miRNA modulation, an increasing number of studies have declared that some circRNAs promoted the proliferation of HCC cells and HCC tumorigenesis through efficient translation. For instance, circβ-catenin was translatable and highly expressed in HCC, and circβ-catenin translation generated a novel isoform named β-catenin-370aa, which subsequently promoted HCC progression by protecting β-catenin from GSK3β-induced degradation and thus facilitated the activation of the Wnt/β-catenin pathway (15).

**Figure 2:**
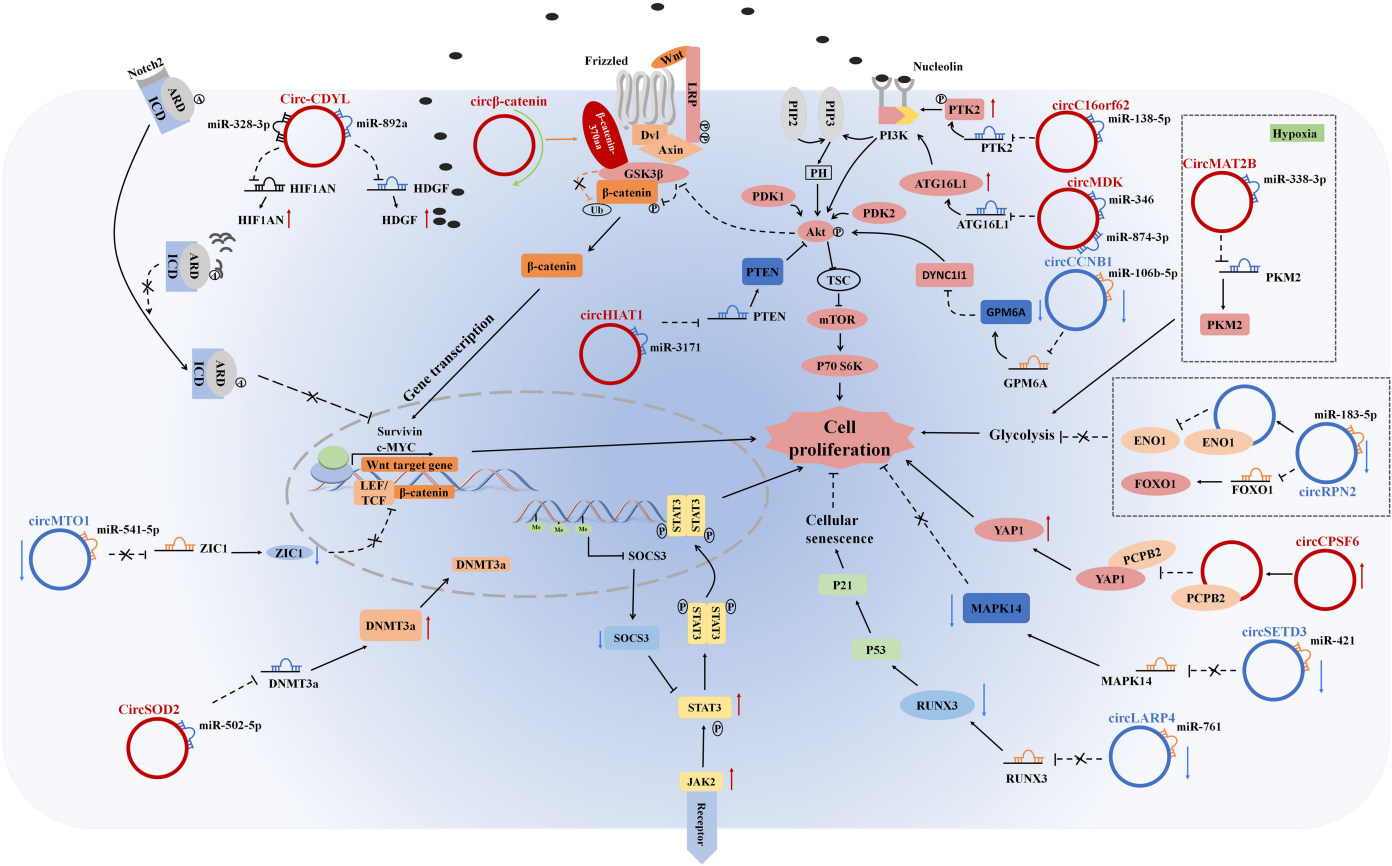
CircRNAs regulate cell proliferation, tumor growth suppression, glycolysis, and cellular senescence in HCC cells by stimulating/inhibiting cell proliferation signaling pathways.

Overall, the above examples clearly demonstrated that circRNAs had crucial regulatory functions in HCC tumorigenesis and progression, contributing to a deeper comprehension of HCC pathogenesis (**Figure 2**).

### 4.2 Apoptosis

Apoptosis, autophagy, and necrosis are three primary mechanisms contributing to programmed cell death that tumor cells are efficient at avoiding (40). Tumor cells can evade apoptosis, enabling cell immortality. STATs are latent transcription factors that participate in cell growth, development, apoptosis, and a series of other cellular events. Increasing evidence has demonstrated that STAT3 plays a critical role in apoptosis (41). As shown in previous studies, human HCC cells constantly stimulated the JAK1/STAT3 pathway to accelerate HCC progression. Circ9119 was a neoteric oncogene that increased cell propagation and inhibited apoptosis through the miR-26a/JAK1/STAT3 signaling pathway (42). Furthermore, m6A-modified circMAP3K4 can be translated into circMAP3K4-455, which decreased cisplatin-induced apoptosis and stimulated HCC progression by directly binding to apoptosis inducing factor mitochondria associated 1 (AIF), thus blocking its nuclear translocation (43). Clearly, circRNAs can regulate the apoptosis of HCC cells.

### 4.3 Cancer stem cells

CSCs have been regarded as the origin of carcinogenesis, chemoresistance, tumor metastasis and recurrence. The Hedgehog signaling pathway plays a critical role in various biological processes including embryonic development, tissue homeostasis and regeneration. Irregular Hedgehog signaling may induce numerous human malignancies and the generation of CSCs. CircIPO11 interacted with TOP1 to trigger GLI1 transcription, thus initiating the self-renewal of liver CSCs and stimulating the development of HCC through mediating Hedgehog signals (44). In addition, CircZNF609 also promoted the proliferation, metastasis, and stemness of HCC cells by mediating the Hedgehog pathway by binding to miR-15a-5p/15b-5p and increasing the GLI2 expression (45). CircZKSCAN1 inhibited stemness by physically interacting with FMRP to prevent FMRP-CCAR1-induced signaling (12). Interestingly, m6A modification of circHPS5 facilitated its cytoplasmic output via an YTHDC1-dependent pattern and accelerated EMT and CSC phenotypes, facilitating tumorigenesis of HCC via the miR-370/HMGA2 axis (46).

### 4.4 Metastasis

Metastasis is characterized by a multistep process that allows tumor cells from the primary tumor to spread into circulation through lymphatic and blood vessels and colonize distant organs, ultimately leading to secondary tumors. MMP9 plays a crucial role in tumor progression and metastasis by degrading extracellular matrix (ECM). Recent studies have demonstrated that circUBAP2 accelerated the progression and metastasis of HCC by functioning as a sponge of miR-194-3p to upregulate MMP9 expression (47). MMP1 had also been demonstrated to play critical roles in migration and invasion in various types of cancers. CircDLC1 inhibited the proliferation and metastasis of HCC through the HuR-MMP1 axis (48). FBLIM1 is a crucial promotor of migration in a wide variety of cell types and is related to more aggressive HCC phenotypes. circFBLIM1 had been reported to promote HCC cell proliferation and invasion by acting as a ceRNA to upregulate FBLIM1 expression (49). SOX9 is a distinguished oncogenic transcription mediator in various human cancers that can activate diverse signaling pathways. Circ-FOXP1 facilitated HCC growth and metastasis via the SOX9/circ-FOXP1/miR-875-3p/miR-421 signaling pathway (50). Emerging evidence had shown that MAPK1 can function as an important tumor promoter in the progression of HCC. Consistent with these findings, circASAP1 accelerated HCC cell proliferation and invasion via miR-326/miR-532-5p MAPK1 signals (51). Focal adhesion kinase (FAK) is a nonreceptor tyrosine kinase which dysregulation had been noted in various types of tumors in relation to tumor metastasis. circRASGRF2 had been confirmed to be sharply increased in HCC and to accelerate HCC progression by sponging miR-1224 to upregulate FAK expression (52). The STAT3 signals is oncogenic and constantly hyperactivated in a variety of human cancers, including HCC. Circ-LRIG3 was notably overexpressed in HCC and functioned as a protein scaffold to promote EZH2-mediated STAT3 methylation and phosphorylation by physically interacting with EZH2 and STAT3. Furthermore, activated STAT3 immediately bound to the circ-LRIG3 promoter region to enhance the transcriptional activity of circ-LRIG3, thereby forming a positive feedback pathway to accelerate the metastasis of HCC (53). Moreover, circRHOT1 accelerated HCC growth and metastasis by recruiting TIP60 to upregulate NR2F6 expression (13).

EMT is an important developmental program for tumor metastasis. Twist1 is a critical EMT-related transcription factor that regulates the expression of EMT-related genes through promoter activation or suppression, inhibiting the transcription of epithelial phenotype-associated genes. For example, Twist1 enhanced vimentin expression and HCC tumorigenesis and metastasis via Twist/circ-10720/vimentin signaling pathway (54). PCBP1 participated in the EMT program through various cancer processes, particularly TGF-β signaling. Importantly, has_circ_0003998 was highly overexpressed in HCC and it participated in EMT via both miR-143-3p/FOSL2 signaling and the PCBP1/CD44v6 axis (55). Moreover, EIF4A3-induced circTOLLIP facilitated the progression of HCC by regulating the miR-516a-5p/PBX3/EMT signaling pathway (56). Hsa_circ_0003288 functioned as an oncogene to facilitate EMT and invasion of HCC by sponging miR-145 and increasing the expression of PD-L1 via the PI3K/AKT signaling pathway (57). The Wnt/β-catenin pathway is a crucial signaling cascade strongly related to tumor progression and its activation facilitates tumor invasion through the upregulation of factors modulating EMT. Circ_0067934, as an oncogene, facilitated HCC development by upregulating the miR-1324/FZD5/Wnt/β-catenin axis (58).

DAB2IP functions as a tumor suppressor for a variety of tumors. circRNA-5692 had been found to inhibit HCC development and EMT progression by accelerating demethylation of the DAB2IP gene and enhancing its expression and circRNA-5692 also acted as a tumor suppressor via the miR-328-5p/DAB2IP pathway (59). EZH2, a histone methyltransferase, is the enzymatically active core subunit of the polycomb repressive complex 2 (PRC2) and is a notable oncoprotein that plays an essential role in malignant cancer behaviors, especially metastasis (60). Circ-ADD3 facilitated the binding of CDK1 and EZH2 by directly interacting with them, leading to subsequent ubiquitination and degradation and ultimately restraining HCC metastasis (61). circTRIM33-12 had been found to be significantly reduced in HCC and functioned as an important tumor suppressor to restrain the proliferation, metastasis and immune evasion of HCC cells by sponging miR-191 and inducing TET1 expression (62). SMAD2, plays a critical role in accelerating EMT progression. However, circSMAD2, originating from SMAD2, was markedly decreased in HCC tissues and restrains the metastasis and EMT of HCC cells by directly interacting with miR-629 (63). Moreover, SMARCA5 acted as a tumor promoter and regulated the Wnt/β-catenin signaling pathway to stimulate the proliferation of HCC cells. However, cSMARCA5, generated by SMARCA5, a tumor suppressor, was notably reduced in HCC and may restrain the growth and metastasis of HCC through the DHX9-cSMARCA5-miR-17-3p/miR-181b-5p-TIMP pathway (64).

Taken together, circRNAs are critical molecular regulators of the EMT process and motility in HCC.

### 4.5 Drug resistance

Chemoresistance is inevitable in HCC treatment. Sorafenib, a multi-target tyrosine kinase inhibitor (TKI), is a first-line treatment for advanced HCC. However, drug resistance consistently appears after long-term application. Numerous factors related to sorafenib resistance in HCC have been demonstrated, such as the Wnt/β-catenin pathway, EMT, the TME and epigenetic regulation (65). Increasing evidence have elucidated that circRNAs were participated in the sensitivity of HCC to chemotherapy. circRNA-SORE was overexpressed in sorafenib-resistant HCC cells and reduced the sensitivity of HCC cells to sorafenib by interacting with miR-103a-2-5p and miR-660-3p; in addition, circRNA-SORE competitively upregulated the Wnt/β-catenin pathway, thereby impairing chemotherapy effectiveness (66). YBX1 is tightly related to tumor progression, chemoresistance, and cancer prognosis. Interestingly, another study demonstrated that circRNA-SORE, which can be delivered extracellularly via exosomes, playing a vital role in prohibiting PRP19-induced YBX1 degradation and was thereby involved in the maintenance and spread of sorafenib resistance in HCC (14). CircFOXM1 was a crucial promotor of sorafenib resistance in HCC that can interact with miR-1324 to stimulate MECP2 expression and therefore strengthened sorafenib resistance in HCC (67). Lenvatinib, another multitarget TKI, had illustrated promising antitumor activity in HCC patients. However, the molecular mechanisms of primary and acquired resistance to lenvatinib remain to be revealed, creating a critical challenge for HCC targeted therapy. CircMED27 promoted lenvatinib resistance of HCC through sponging miR-655-3p and increasing the expression of USP28 (68). CircKCNN2, transcriptionally inhibited by NFYA, suppressed HCC recurrence and associated with lenvatinib resistance by reducing the FGFR4 expression through the miR-520c-3p/MBD2 axis (69).

In addition to sorafenib and lenvatinib, cisplatin is one of the most universally applied chemotherapy drugs to cure advanced HCC. However, acquired multidrug resistance (MDR) in response to cisplatin treatment remains the biggest obstacle to an ideal therapeutic effect in HCC patients (70). Indeed, circARNT2 was highly expressed in cisplatin-resistant HCC tissues and functions as a tumor promotor by inhibiting miR-155-5p, leading to PDK1 overactivation and eventually resistance to cisplatin in HCC cells (71). Another study revealed that circMRPS35 exerted its oncogenic function in HCC not only by sponging miR-148a to modulate the STX3-PTEN pathway, but also by being translated into circMRPS35-168aa, resulting in cisplatin resistance, which indicated that circMRPS35 could be a crucial factor in the progression and chemoresistance of HCC with different expression patterns under different conditions (16).

In summary, emerging reports have illustrated that circRNAs play a crucial role in the drug resistance of HCC. However, research on the role of circRNAs in chemoresistance is still at the early stage, and we must further elucidate the chemoresistance mechanism of countless circRNAs in HCC. Moreover, future translational studies and/or clinical trials are necessary to develop circRNA-targeted treatments, which may ultimately improve the prognosis of HCC patients by enabling patients to gradually overcome drug resistance.

### 4.6 Angiogenesis

Tumor angiogenesis, which is important for cancerous growth, continuously supplies malignant tissues with necessary oxygen and nutrients and removes metabolic wastes, playing an essential role in tumor progression and development. Without angiogenesis, tumors cannot grow beyond a size of 1-2 mm (72). FGF signaling pathways are some of the most potent stimulants of angiogenesis and can facilitate the proliferation, migration and differentiation of endothelial cells in culture. During hepatocarcinogenesis, FGFR1 disrupts DNA synthesis and cell proliferation at an early stage and further boosts neoangiogenesis at later stages, and these processes are mediated by vascular endothelial growth factor. According to Zhan et al. the hsa_circ_103809/miR-377-3p/FGFR1 axis may play an essential role in the development of HCC (73).

### 4.7 Tumor microenvironment

The TME is dependent on the crosstalk between various cell types, especially Cancer-associated fibroblasts (CAFs), ECM cells and infiltrating immune cells and plays a crucial role in the progression, metastasis and therapeutic treatment of cancer (74). A growing amount of evidence has revealed the intricate relationship between circRNAs and critical components in the TME.

Tumor cells develop and metastasize through a variety of mechanisms to evade recognition and attack by the immune system, a phenomenon known as tumor immune escape. Immunosuppression is one of the most vital mechanisms of tumor immune escape. Snail is a transcriptional mediator of DPP4 that promotes local immunosuppression by decreasing lymphocyte infiltrating via decreasing the expression of the chemokine CXCL10. A recent report claimed that circMET acted as a novel onco-circRNA that stimulated HCC progression and immune escape through the snail/DPP4/CXCL10 signaling pathway (75).

Natural killer (NK) cells are regarded as the major cell involved in host immune surveillance and play a substantial role in antitumor immunotherapy. Growing evidence illustrated that circRNAs secreted by tumor cells played an essential role in tumor immune surveillance by strengthening NK-cell activity and enhancing NK-cell-mediated immune surveillance. For instance, circARSP91 was found to enhance the ability of NK cells to recognize and attack target tumor cells by interacting with ULBP1 to increase the expression of ULBP1 (76). However, hsa_circ_0007456 may extremely impair HCC cells susceptibility to NK cells via the miR-6852-3p/ICAM-1 signaling pathway (77). Tumor-associated macrophages (TAMs), especially TAMs with the M2 phenotype are the most crucial immune cells in the TME that stimulate tumor progression and metastasis. CircASAP1 facilitated macrophage proliferation and chemotactic migration by sponging miR-326 and miR-532-5p and thus inducing overexpression of CSF-1, which was a crucial regulator of macrophage differentiation and function, resulting in macrophage recruitment to the tumor bed (51). CCL2, as a chemokine, bind to C-C motif chemokine receptor 2 (CCR2) and recruited monocytes and macrophages, and CCL2 was regulated by the activation of PPARα. Hsa_circ_0110102 regulated the miR-580-5p/PPARα/CCL2 signaling pathway in HCC. Interestingly, CCL2 released into the TME mediated the expression and delivery of COX-2 and PGE2 in macrophages to stimulate the proliferation of HCC cells (78). Moreover, hsa_circ_0003410 facilitated HCC progression by increasing the ratio of M2/M1 macrophages through stimulating the expression of CCL5 (79).

In addition, CAFs play an important role in tumor progression by modulating the inflammatory microenvironment. CXCL11 functioned as a critical regulator in regulating the interaction between CAFs and CAF-adjacent cancer cells and served as an extracellular remodeler to facilitate HCC cell migration and metastasis by upregulating the circUBAP2/miR-4756/IFIT1/3 pathway (80).

**Figure 3:**
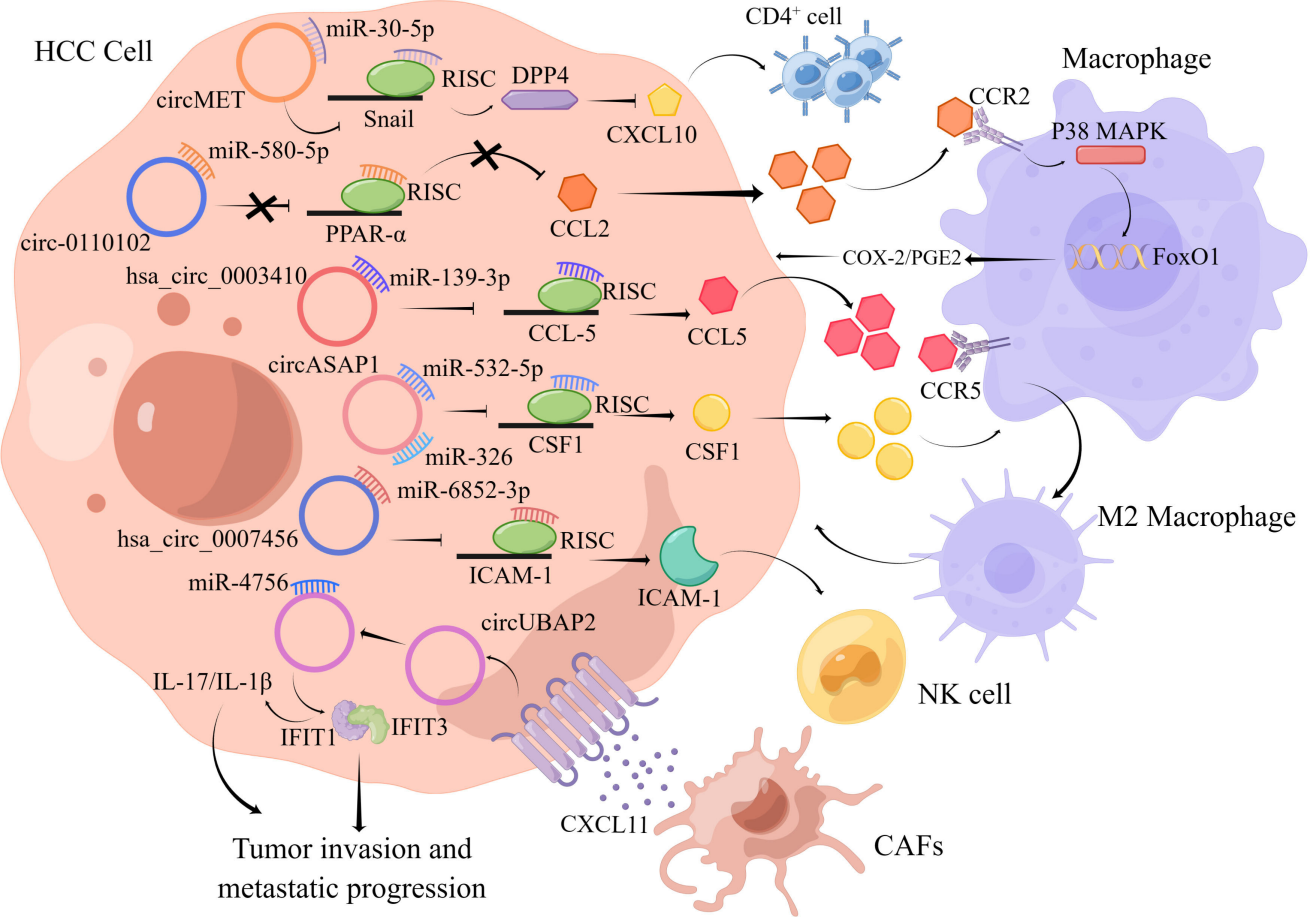
The figure illustrates the interaction between circRNAs and important components in the HCC tumor microenvironment.

Growing evidence suggested that circRNAs could participate in HCC progression by affecting the immune system of HCC patients (**Figure 3**). However, research on circRNAs in the TME is still in its infancy, and the specific roles of circRNAs in the TME remain to be further explored.

### 4.8 Exosomes

Exosomes are small extracellular vesicular bodies with a diameter of 30-100 nm that are released from a variety of cells and contain mRNAs, miRNAs, circRNAs and proteins that may affect various biological functions of recipient cells (81). Emerging evidence have revealed that exosomes exert a crucial function in tumor development processes, including angiogenesis, metastasis, drug resistance and TME processes. Recently, exosome-derived circRNAs have been proven to be a potential factor related to tumorigenesis and progression. Evidence provided that exosomal circPTGR1 from highly metastatic cells could have a notable impact on cell metastatic potential by restraining the miR449a-MET interaction in recipient cells, resulting in lethal damage in TME homeostasis and accelerating HCC progression (82). In another study, circ_MMP2 was found to be transported by 97-Hderived exosomes stimulated HCC metastasis by sponging miR-136-5p to enhance the expression of its target gene, MMP2 (50). Tumor cell-derived exosomal circ-0072088 promoted the migration and invasion of HCC cells by sponging miR-375 and upregulating MMP-16 (83). Notably, circRNA-SORE was delivered by exosomes, which promoted transmission of sorafenib resistance among HCC cells (14).Moreover, exosomal circ-0004277 directly derived from HCC cells can be delivered to normal surrounding cells and may modulate their biological functions by suppressing the expression of ZO-1 and stimulating the development of EMT (84). In another study, circRNA Cdr1as functioned as a ceRNA to accelerate the development of HCC by inhibiting miR-1270 to increase AFP expression. In addition, the exosomal circRNA Cdr1as secreted by HCC cells, can be delivered to surrounding normal cells to ultimately promote the malignant processes of peripheral normal cells (85).Moreover, exosome circ-DB generated from adipocytes was increased in HCC patients with higher body fat values. Moreover, researchers have illustrated that exo-circ-DB increased HCC progression and reduced DNA damage by reducing the level of miR-34a and activating the deubiquitination-related USP7/cyclin A2 signaling pathway (86).

Interestingly, exosomes may generate an immunosuppressive environment favoring tumor cell survival by serving as messengers between tumor cells and peripheral recipient cells. HCC-secreted exosomal circUHRF1 increased the expression of the miR-449c-5p target gene TIM-3 in NK cells by absorbing miR-449c-5p, and thus stimulated immune escape and reduced sensitivity to anti-PD1 immunotherapy in HCC (87). HCC-derived exosomal circTMEM181 sponged miR-488-3p and elevated the expression of the CD39 in macrophages and the expression of the CD73 in HCC cells, which collaboratively stimulated the eATP-adenosine pathway and generated more adenosine, damaging CD8^+^ T cell function to indulge immunosuppression and acquire anti-PD1 resistance in HCC (88). Moreover, exosomal circGSE1 exerted tumor immunosuppressive functions by inducing the expansion of Tregs via activation of the miR-324-5p/TGFBR1/Smad3/FOXP3 signaling pathway (89). Interestingly, hsa_circ_00074854 may regulate the growth and metastasis of HCC cells by enhancing HuR protein stability and activating the ZEB1 axis. In addition, HCC cell-derived hsa_circ_00074854 may be transmitted to macrophages by exosomes and stimulated macrophage polarization toward the M2 type, which facilitated the metastasis and EMT of HCC cells (90). In addition, exosomal hsa_circ_0004658 secreted by RBPJ (recombination signal binding protein-Jκ) -overexpressing macrophages inhibited HCC progression through the miR-499b-5p/JAM3 pathway (91).

**Figure 4:**
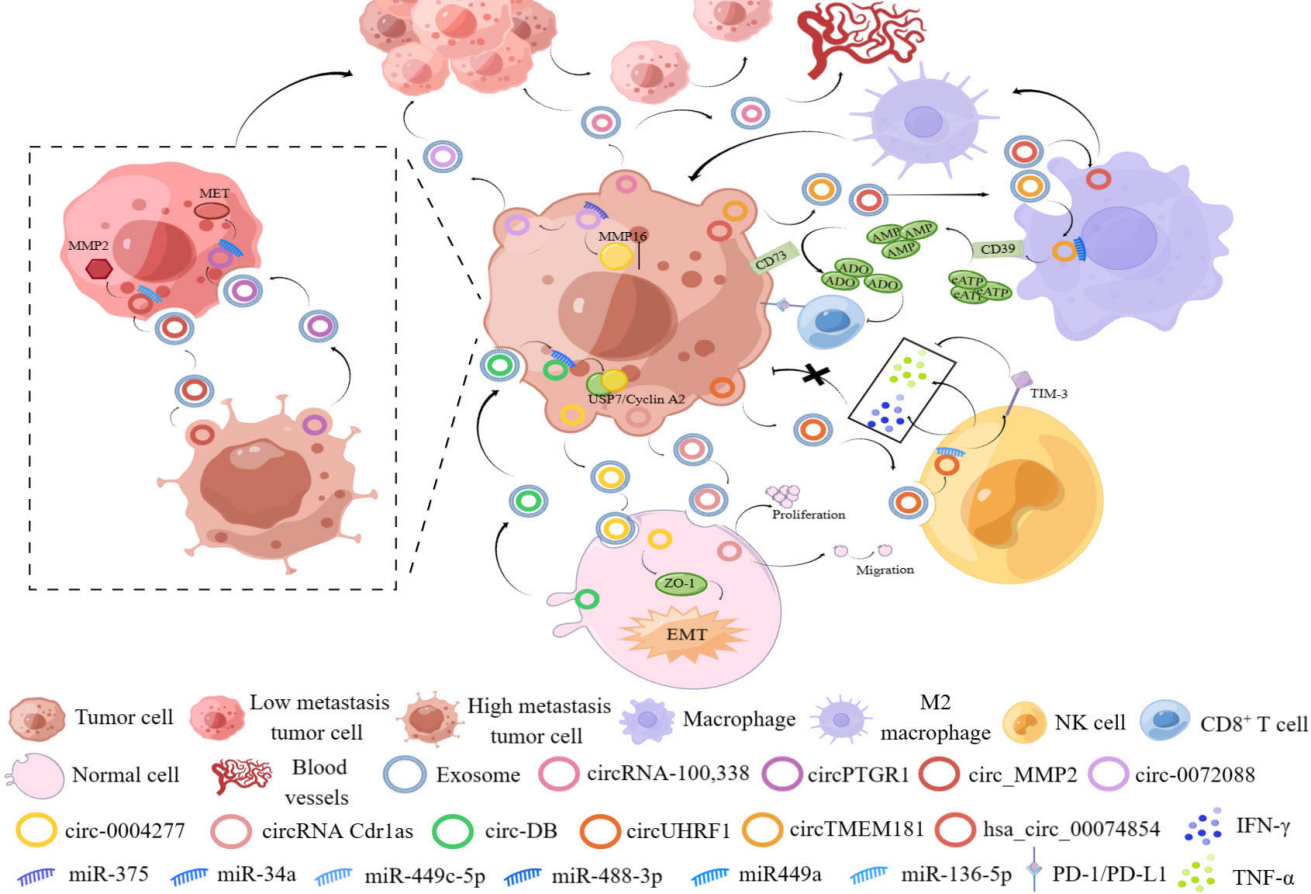
The figure shows the important role of exosomal circRNAs from different sources in the occurrence and development of HCC.

This evidence confirmed that exosomal circRNAs secreted by HCC cells, normal cells and immune cells exert critical functions in HCC progression, metastasis and drug resistance, suggesting that it is important to study the molecular mechanisms enabling exosomal circRNAs from different secreted sources to play an important role in HCC progression (**Figure 4**).

## 5 Clinical significance of circRNAs in HCC

Due to the lack of obvious symptoms at the early stage and lack of dependable and valid biomarkers, HCC patients are frequently diagnosed at the advanced stage and the overall survival (OS) rate is less than ideal. Furthermore, current diagnostic biomarkers, including α-fetoprotein (AFP), α-fetoprotein-L3 (AFP-L3), and des-carboxy-prothrombin (DCP), show poor sensitivity in diagnosing HCC. CircRNAs are closely related to various biological processes in HCC, and circRNAs are different from their homologous linear RNAs, as they have a distinctive closed-loop construction structure that aids detection in tissues, saliva, plasma, serum, urine, exosomes and other biological samples. Furthermore, circRNAs have been proposed as novel early- stage diagnostic and prognostic biomarkers for HCC because of their abundance, stability, conservation nature and specificity in tissues and cells.

### 5.1 CircRNAs as diagnostic biomarkers for HCC

Emerging evidence utilizing clinical HCC samples have illustrated the aberrant expression, increased disease specificity and clinical significance of specific circRNAs, suggesting that they are attractive biomarker candidates for HCC diagnosis (**Table 1**). For example, circZNF566, which functioned as a tumor promoter, was increased significantly in HCC tissues. The AUC of circZNF566 was 0.834, with a sensitivity and specificity of 82.2% and 72.4%, respectively, showing that circZNF566 was a potential biomarker for the clinical diagnosis and evaluation of HCC (92). The AUC of circRNA_104075 for HCC diagnosis was 0.973, and the sensitivity and specificity were 96.0 and 98.3%, respectively, indicating remarkable diagnostic value (93). The AUC of hsa_circ_0091570 in diagnosing HCC was 0.736, suggesting that it could be used as a diagnostic biomarkers (94). CircCRIM1 was significantly elevated in HCC tissues and was related to large tumor size and advanced TNM stage and Edmondson grade. Thus, circCRIM1 was a potential prognostic biomarker for HCC (95). In addition to HCC tissue, serum exosomes can also be assessed to reveal expression changes of circRNAs as diagnostic biomarkers for HCC. Compared with nonmetastatic HCC samples, samples from HCC patients with pulmonary metastasis showed notable increases in exosome-derived circRNA-100338 in serum (96). Moreover, plasma hsa_circ_0005397 combined with serum AFP and AFP-L3 had more promising diagnostic performance (97).

**Table 1:** The potential circRNAs as diagnostic biomarkers in HCC.

CircRNA	Dysregulation	Samples	AUC	Sensitivity (%)	Specificity (%)	Clinicopathological association	PMID
circ_104075	up	Tissue and serum	0.973	96	98.3	–	30361504
circ‐CDYL(+HDGF+HIF1AN)	up	Tissue	0.73	75.36	66.67	–	31148183
circRASGRF2	up	Tissue	0.882	81.4	95	tumor size, tumor differentiation, tumor stage, microvascular invasion	33312757
circ-LRIG3	up	Tissue	0.8681	78.43	95.19	–	33222697
hsa_circ_0005397	up	serum	0.737	82	58.8	tumor size, TNM stage	33679420
circ-0072088	up	Tissue and serum	0.899	–	–	Tumor size, the number of nodules, Edmondson grade, TNM stage,	34568335
							34148288
CircCRIM1	up	Tissue	–	–	–	Tumor size; TNM stage; Edmondson grade	34869393
circ-ZEB1.33	up	Tissue and serum	–	–	–	TNM stage	30123094
CircRNA Cdr1as	up	Tissue	–	–	–	tumor diameter, AFP, tumor satellite	
circ-FOXP1	up	Tissue and serum	0.9318			tumor size, TNM stage, microvascular invasion	31698267
circZNF566	up	Tissue	–	–	–	tumor size, tumor differentiation, M stage,	32532962
circGFRA1	up	Tissue	–	–	–	tumor size, intrahepatic metastasis, extrahepatic metastasis, BCLC stage and TNM stage	33431945
circWHSC1	up	Tissue and serum	–	–	–	–	33410156
circRHBDD1	up	Tissue	–	–	–	Tumor number, microvascular invasion, tumor size, α-fetoprotein, TNM stage	35317519
circ_0003945	up	Tissue	–	–	–	tumor size, CNLC stage	35170199
has-circ-0000221	up	serum	–	–	–	–	35057034
circ-ADD3	down	Tissue	0.8878	–	–	Vascular invasion, Intrahepatic metastasis, Distant metastasis	31497351

### 5.2 CircRNAs as prognostic biomarkers for HCC

CircRNAs can function as biomarkers to predict patient survival parameters as well, including overall survival (OS), disease-free survival (DFS), and progression-free survival (PFS). To evaluate the prognostic function of circRNAs in HCC, we gathered data from reporter and analyzed the connections between circRNA expression and OS, DFS, and PFS (**Table 2**). Twenty increased circRNAs and eleven decreased circRNAs were illustrated to predict poor OS. Upregulated circMAP3K4, circRHBDD1 and circTOLLIP expressions were related to poor prognosis implying that these circRNAs were prospective prognostic markers for HCC (26, 43, 56). Furthermore, downregulated expression of circUBE2J2 and circKCNN2 can serve as independent predictors of poor OS in HCC (69, 98).

**Table 2:** The potential circRNAs as prognostic biomarkers in HCC.

CircRNA	Dysregulation	Prognosis	Univariate Analysis			Multivariate Analysis			PMID
			HR	95% CI	p	HR	95% CI	p	
circMAP3K4	Up	OS	1.662	1.083-2.549	0.02	–	–	–	35366894
		DFS	1.733	1.130-2.656	0.012	–	–	–
circRHBDD1	Up	OS	1.549	1.046-2.293	0.029	–	–	–	35317519
		DFS	2.388	1.417-4.024	0.001	–	–	–
circUBAP2	Up	OS; RFS	–	–	–	–	–	–	34239873
CircCRIM1	Up	OS	–	–	–	–	–	–	34869393
CircRNA-104718	Up	OS	–	–	–	–	–	–	31278132
circRHOT1	Up	OS; RFS	–	–	–	–	–	–	31324186
Exo-circPTGR1	Up	DFS	–	–	–	–	–	–	30630697
circ-FOXP1	Up	OS	–	–	–	–	–	–	31698267
circADAMTS13	Up	RFS	–	–	–	–	–	–	30537115
circASAP1	Up	OS	–	–	–	–	–	–	31838741
circZNF566	Up	OS, DFS	–	–	–	–	–	–	32532962
circRASGRF2	Up	OS	–	–	–	–	–	–	33312757
circC16orf62	Up	OS	–	–	–	–	–	–	34108451
circ-LRIG3	Up	OS, DFS	–	–	–	–	–	–	33222697
circ_MMP2	Up	OS	–	–	–	–	–	–	31944556
circGFRA1	Up	OS	–	–	–	–	–	–	33431945
circWHSC1	Up	OS	–	–	–	–	–	–	33410156
hsa_circ_0005397	Up	OS	–	–	–	–	–	–	33679420
circ0013958	Up	OS	–	–	–	–	–	–	33937016
circTOLLIP	Up	OS, DFS	–	–	–	–	–	–	35509064
hsa_circ_0005986	Down	OS	0.504	0.307–0.828	0.007	0.572	0.339–0.966	0.037	34294754
		PFS	0.492	0.317–0.763	0.002	0.573	0.362–0.906	0.017
circUBE2J2	Down	OS	0.3337	–	0.0117	0.4106	–	0.0451	34686662
circKCNN2	Down	OS,	–	–	0.013	–	–	–	35051313
		RFS	–	–	0.011	–	–	–
circLARP4	Down	OS	–	–	–	3.997	1.747‐9.142	0.001	30520539
		PFS	–	–	–	2.347	1.119‐4.923	0.024
circDLC1	Down	OS, RFS	–	–	–	–	–	–	33391541
circMTO1	Down	OS	–	–	–	–	–	–	28520103
circ-102, 166	Down	OS; RFS	–	–	–	–	–	–	33034848
circSETD3	Down	OS; RFS	–	–	–	–	–	–	30795787
circ-ADD3	Down	OS, RFS	–	–	–	–	–	–	31497351

In summary, the abnormal expression of circRNAs can be monitored not only in tumor tissues but also in body fluids, such as blood, saliva and urine, as well as in exosomes in these fluids, suggesting that they are valuable noninvasive biomarker candidates. However, there are still many challenges ahead in exploring circRNAs as biomarkers for the clinical diagnosis and prognostication of HCC, especially those derived from blood and urine. In another way, a single circRNA class is unlikely to be able to predict HCC progression or therapy effects, as the expression may vary per patient and the heterogeneity of tumors in different patients.

## 6 The clinically translational value of circRNAs in HCC treatment

In the emerging world of RNA drugs, circRNA, as a novel multifunctional therapeutic target that can transmit genetic information, is expected to be a potential alternative to mRNA due to its excellent stability. As mentioned above, circRNAs are more stable than mRNA and specific express in tissue or cell type which may exert potential therapeutic targets in HCC. Results of previous study illustrated that some potential treatments such as RNAi vectors, conditional circRNAs knockout or knockdown through the cre-lox system, CRISPR/Cas9 and CRISPR/Cas13 system (99). A troublesome in utilizing circRNAs as therapeutic targets is the conveyance of the circRNA-targeted-siRNA or circRNA overexpressed vector *in vivo*. In one way, exosomes are being explored as delivery platforms for circRNA-targeted agents. As previously mentioned, some exosome-circRNAs can facilitate the transformation of the tumor microenvironment and promote or suppress the invasion and drug resistance of HCC. However, low loading efficiencies and low exosome yields are capital challenge to overcome when using exosomal circRNAs for the treatment of HCC. Interestingly, in another way, the use of nanoparticles (NPs) as a delivery platform has dramatically enhanced the feasibility of circRNA-targeted treatment *in vivo*. The PAE-based si-circMDK nanoparticles effectively inhibited HCC proliferation and metastasis *in vivo* (18). PLGA-PEG (si-circROBO1) NPs exhibited excellent anti-HCC activity *in vitro* and *in vivo* (100). Lipid nanoparticles (LNPs) are clinically advanced carriers for delivering RNA to target organs. Since most LNPs have a propensity to accumulate in hepatocytes and limit their use to targets outside of the liver (101). This limitation may be of great aid in the delivery of circRNAs to the liver for the treatment of HCC. However, the treatment methods for circRNA are still in the preliminary stage, further clinical trials investigating the feasibility of them are required.

## 7 Conclusion and future perspectives

Initially, circRNAs were regarded as the results of random transcriptional RNA splicing errors in transcription. With the rapid advancement of high throughput sequencing technologies and bioinformatics tools, growing evidence has illustrated that numerous aberrantly expressed circRNAs have a strong correlation with the progression of HCC. Some differentially expressed circRNAs are correlated with the clinicopathological features in HCC patients. Moreover, aberrant circRNA expression exists, and increases in the expression of some circRNAs are related to the tumorigenesis, proliferation, metastasis, apoptosis and drug resistance of HCC. Accumulating studies have demonstrated the biological function of circRNAs, including miRNA sponges, RBP sponges, regulators of gene transcription and potential regulators of translation. However, the comprehensive biological functions and specific roles of circRNAs in HCC occurrence and development remain uncertain.

Novel insights into circRNAs have emerged rapidly. However, circRNAs in HCC are still relatively underexplored compared with miRNAs and lncRNAs in HCC and most studies have been cross-sectional association studies. Furthermore, the mechanisms of circRNA formation, cellular localization and degradation are still not clear. In addition, only little critical circRNAs in HCC have been distinguished and characterized. Innovative roles of circRNAs in addition to their role as miRNA sponges need to be revealed. Notably, recent studies have point to the importance of using tumor tissues, perhaps the exploration of circRNAs in tumor tissues is a more intuitive reflection of HCC progression than tumor cell lines. In the future, studies detecting the level of circRNAs in body fluids and analyzing the sensitivity and specificity of such circRNAs as biomarkers are needed. The ultimate purpose of various circRNA studies is to enable the application of circRNA in early clinical diagnosis and early treatment, and many experiments are still needed to accomplish this goal.

## Author contributions

Z-DL and CZ wrote, revised and edited the manuscript. Z-DL, JL and SL prepared all tables and figures. Y-LL and L-HZ supervised the study. All authors participated in the design of the review and revised the manuscript. All authors have read and approved the final manuscript.
